# A Novel Design Approach for Self-Crack-Healing Structural Ceramics with 3D Networks of Healing Activator

**DOI:** 10.1038/s41598-017-17942-6

**Published:** 2017-12-19

**Authors:** Toshio Osada, Kiichi Kamoda, Masanori Mitome, Toru Hara, Taichi Abe, Yuki Tamagawa, Wataru Nakao, Takahito Ohmura

**Affiliations:** 1 National Institute for Materials Science, 1-2-1 Sengen, Tsukuba, Ibaraki 305-0047 Japan; 2 National Institute for Materials Science, 1-1 Namiki, Tsukuba, Ibaraki 305-0044 Japan; 3 Yokohama National University, 79-5 Tokiwadai, Hodogaya, Yokohama 240-8501 Japan

## Abstract

Self-crack-healing by oxidation of a pre-incorporated healing agent is an essential property of high-temperature structural ceramics for components with stringent safety requirements, such as turbine blades in aircraft engines. Here, we report a new approach for a self-healing design containing a 3D network of a healing activator, based on insight gained by clarifying the healing mechanism. We demonstrate that addition of a small amount of an activator, typically doped MnO localised on the fracture path, selected by appropriate thermodynamic calculation significantly accelerates healing by >6,000 times and significantly lowers the required reaction temperature. The activator on the fracture path exhibits rapid fracture-gap filling by generation of mobile supercooled melts, thus enabling efficient oxygen delivery to the healing agent. Furthermore, the activator promotes crystallisation of the melts and forms a mechanically strong healing oxide. We also clarified that the healing mechanism could be divided to the initial oxidation and additional two stages. Based on bone healing, we here named these stages as inflammation, repair, and remodelling stages, respectively. Our design strategy can be applied to develop new lightweight, self-healing ceramics suitable for use in high- or low-pressure turbine blades in aircraft engines.

## Introduction

Lightweight, self-healing ceramics would greatly increase fuel efficiency in aircraft. For example, structural ceramics with high specific strength and rigidity, and excellent heat resistance are strong candidates for use as turbine blades in aircraft engines, particularly given the industry-wide focus on fuel efficiency^1–3^. Recent damage-resistant ceramics^4–8^ are optimised for high-temperature applications and have reinforcing hierarchical architectures^4–9^. However, even these advanced ceramics remain unsuitable for use in turbine blades, which have strict safety requirements. Catastrophic foreign object damage and consequently unpredictable lifespan limit the application of most monolithic ceramics in high-speed rotating blades operating at high temperatures^10^.

Strategies that confer self-healing properties may extend the applications of ceramic materials. Notably, biomechanical structures, including compact bones in humans, are long-lived and highly reliable, albeit brittle components. This reliability is attributed not only to reinforcing architectures, but also to their self-healing ability and capacity for full recovery from injuries^11,12^. Self-healing consists of ordered but overlapping stages, including inflammation, repair (soft callus formation), and remodelling (hard callus formation and remodelling)^12^. Circulating blood completely fills gaps and cracks in injured bone during inflammation and recruits osteoclasts and osteoblasts for bone regeneration. In addition, necessary elements are delivered via networks of capillary blood vessels and lacuno-canalicular networks of osteocytes.

Recently, there have been many attempts to imitate self-healing^13,14^ in structural materials, such as polymers^15^, concrete^16^, and ceramics^17–26^. In high-temperature structural ceramics, self-healing is often achieved by oxidation reactions that occur at high temperatures or that exploit environmental conditions^17–26^. In this approach, a non-oxide ceramic with high strength and high oxidizability in the environment in which it is used, referred to as a healing agent, is compounded into the oxide ceramic matrix. In this system, the non-oxide healing agent is protected from oxidation by the oxide ceramic matrix. Damage exposes the healing agent to high temperatures or to the atmosphere, thus triggering an oxidation reaction that fills and bonds the damaged surface, allowing autonomous, complete recovery. This strategy has been implemented using silicon carbide (SiC)^17–25^, compounded MAX phases^26^, and others.

However, this mode of self-healing mimics only the inflammation stage of bone repair, and depends on healing agents with a constant and high reaction rate. We have now enhanced the already excellent self-healing capacity of an Al_2_O_3_/SiC composite ceramic^19–23,25^ by producing a mobile phase enabling efficient oxygen delivery, and by incorporating an additional network of healing activator. This approach significantly accelerates fracture gap filling and promotes regeneration of a mechanically strong crystal phase. We also investigated the potential application of this approach in fabricating ceramics for use as turbine blades or other components in aircraft engines.

## Results and Discussion

### Acceleration of strength recovery by a network of healing activator

We have fabricated a new bioinspired self-healing ceramic infused with a three-dimensional (3D) network of healing activator. The healing activator was selected based on thermodynamics as described in subsequent sections (and in supplementary data). The base material was an alumina (Al_2_O_3_) ceramic containing 30 vol% SiC. Al_2_O_3_/SiC composites that effectively self-heal at high temperatures, even with constant or cyclic^20,21^ stress loading and at low partial pressures of oxygen^22^. In addition, the self-healed parts resist high temperatures^19,23^, constant stress, and cyclic fatigue^19^ better than the base material. Elucidation of the underlying healing mechanism is expected to enable further enhancement of material properties.

**Figure 1:**
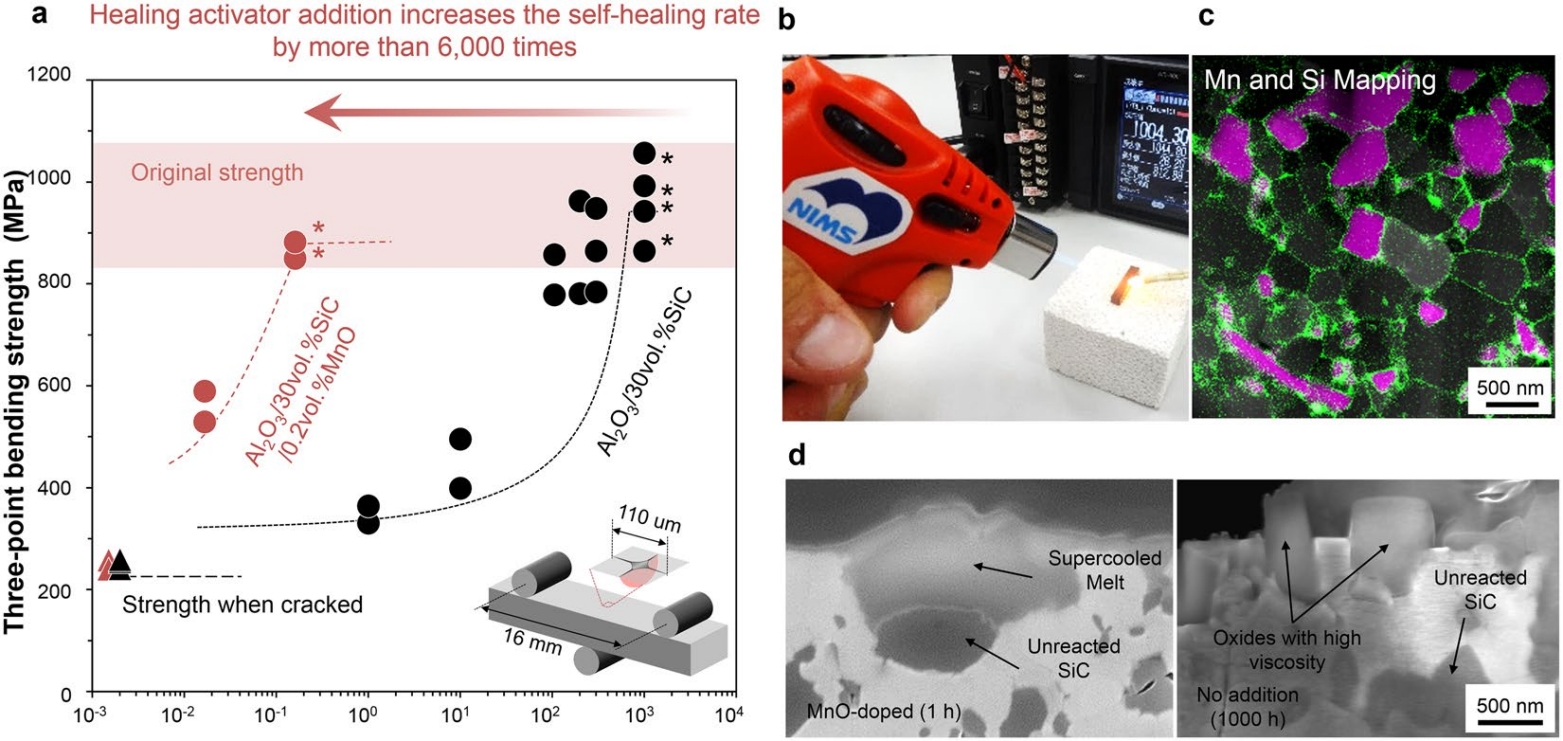
Self-healing and strength recovery in Al_2_O_3_ ceramics containing 30 vol.% SiC and doped with or without 0.2 vol.% of the healing activator MnO. (**a**) Drastically increased rate of strength recovery at 1,273 K. Asterisks indicate specimens that fractured during three-point bending at sites other than the healed Vickers indentation crack. (**b**) Complete self-healing with a gas lighter. (**c**) Localization of the healing activator MnO in a 3D network of Al_2_O_3_ grain boundaries and SiC/Al_2_O_3_ interfaces. Green and red indicate elementally mapped Mn and Si, respectively. (**d**) Surface oxides in the presence or absence of MnO.

Complete healing depends on the production of oxides in amounts sufficient to fill the damaged site. The minimum time to complete healing, *t*_min_, depends on the oxide production rate and increases exponentially as the temperature decreases (Fig. S1). For example, a surface crack with a surface length of 110 μm introduced by Vickers indentation heals in 1,000 h at 1,273 K, indicating that healing is impractical at this temperature (Fig. 1a). In contrast, the same material doped with 0.2 vol% MnO rapidly heals at 1,273 K, and returns to full strength within 10 min, suggesting that even a small amount of MnO, a healing activator, accelerates healing 6,000-fold, and enables complete damage repair even with a simple ignition device, e.g., a gas lighter (Fig. 1b), the maximum temperature of which is equal to the temperature of gas at the front blades of a low-pressure turbine in an aircraft engine. Importantly, MnO doping does not compromise the original strength and cracked strength. Furthermore, materials with and without doping had almost the same fracture toughness: 3.2 MPa·m^1/2^ and 3.4 MPa·m^1/2^, respectively.

Materials with or without MnO are similar in SiC content and particle size, particle size distribution, alumina grain size, bending strength, fracture toughness, and other characteristics (see Fig. S2a,b). Therefore, the healing rate in both materials should be equal if healing is due only to the high-temperature oxidation of SiC according to the following reaction:

1 $${\rm{SiC}}({\rm{s}})+\frac{3}{2}{{\rm{O}}}_{2}({\rm{g}})={{\rm{SiO}}}_{2}({s})+\mathrm{CO}({\rm{g}})$$

The two materials differ in the presence of Mn-rich networks in the initial microstructure and the shape of the healing material after healing. In the presence of a Mn-rich phase on Al_2_O_3_ grain boundaries (also see Fig. S2c–g) and Al_2_O_3_-SiC interfaces (Fig. 1c), the oxide resembled a water droplet, whereas in the absence of MnO, it was deposited in column-like structures (Fig. 1d). Presumably, this is because the supercooled oxide melt in the presence of MnO does not exceed a certain viscosity and undergoes viscoelastic deformation depending on the wettability of melts on fracture surfaces. The low-viscosity supercooled melt, being mobile, helps to fill gaps, similar to blood in fractured bone. These results indicate that the viscosity of the healing agent significantly affects the healing rate.

### Mechanism of self-healing

Insights for the rational design of new materials with a healing activator phase were obtained by investigating the healing mechanism of Al_2_O_3_/30 vol.% SiC. Even in MnO-free materials, oxides shaped like water droplets are observed at approximately 1,473 K (Fig. S1), at which point rapid and complete healing can be achieved^18^. However, the glass transition temperature *T*_g_ of pure SiO_2_ is approximately 1,500 K, suggesting that the supercooled oxide melt is not pure SiO_2_^27,28^. In addition, pure SiO_2_ does not crystallise readily and is a strong glass with a very stable amorphous structure^28,29^, which is inconsistent with the observed resistance to high-temperature fatigue stress^19^. Thus, we hypothesized that the Al_2_O_3_ matrix dissolves to SiO_2_ during healing to form a low-viscosity supercooled aluminosilicate healing material^30–32^. Incorporation of alumina may destabilize the structure of SiO_2_ glass and promote crystallisation^30,31^.

**Figure 2:**
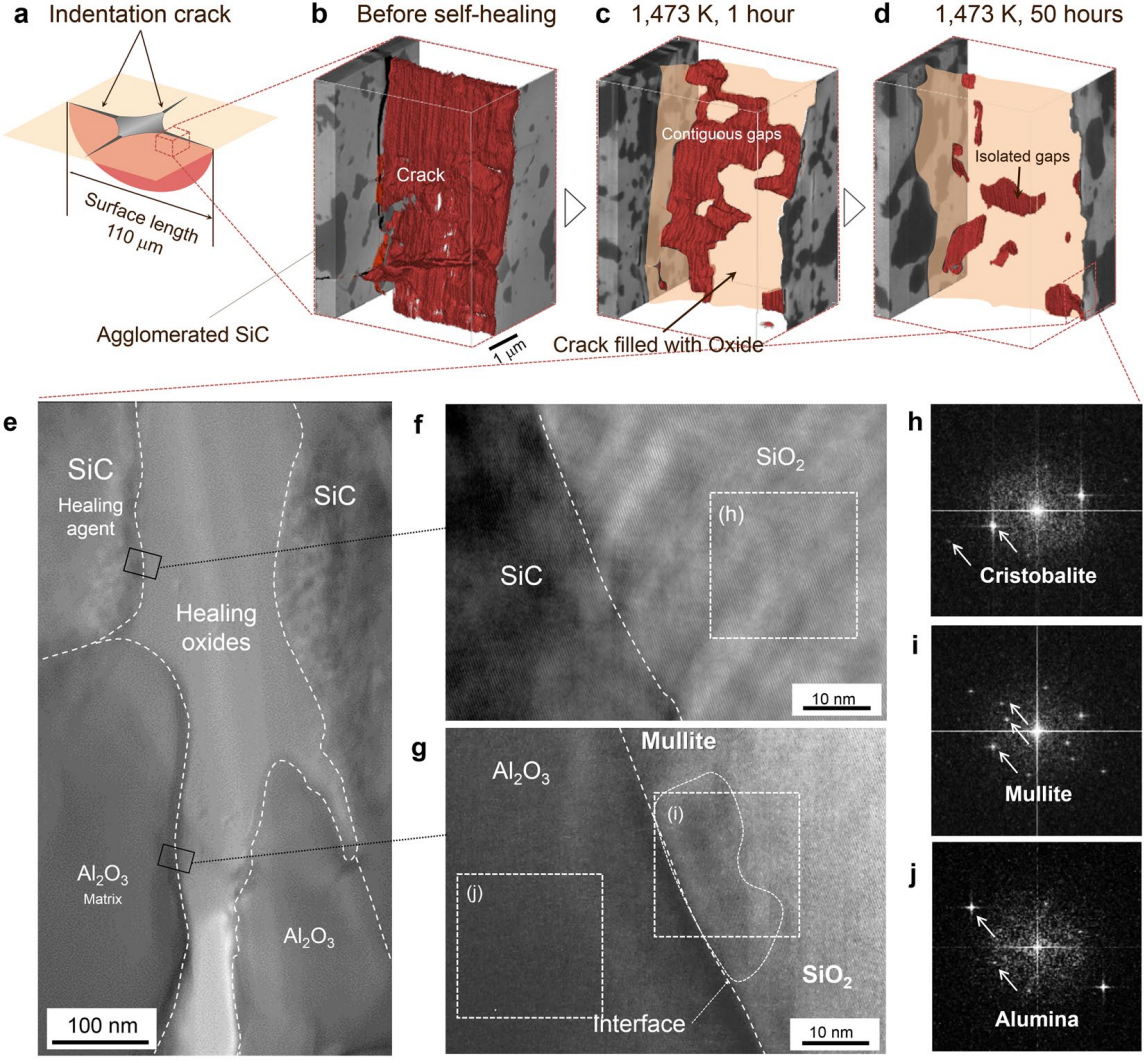
Structure of gaps in Al_2_O_3_/SiC composites healed at 1,473 K. (**a**–**d**) 3D reconstructions of the largest gap before (**a**) and after healing (**b**) at 1,473 K for 1 (**c**) and 50 hours (**d**). (**e**–**j**) High-resolution transmission electron microscopy of healed cracks (**e**), of the interface between SiC and SiO_2_ (**f**), and between Al_2_O_3_ and SiO_2_ (**g**). Fast Fourier transform patterns indicating cristobalite (**h**), mullite (**i**), and alumina (**j**) at sites marked h, i, and j, respectively, in **f** and **g**.

To test this hypothesis, we analysed the 3D structure of a damaged site in Al_2_O_3_/SiC (Fig. 2a). A Vickers indentation crack in this material propagates mainly along the alumina grain boundary and the alumina/agglomerated SiC interface, with an opening of approximately 150 nm near the indent tip (Fig. 2b). After healing at 1,473 K for 1 h without recovery to full strength (Fig. S1a), the exposed SiC (mean particle size 0.27 μm) oxidised and partially bonded or bridged the cracked surfaces (Fig. 2c). However, the amount of healing material was insufficient, and bridging occurred mainly in sites where agglomerated SiC (2–5 μm) was present. Consequently, unrepaired defects were large and contiguous in three dimensions, resulting in a loss of strength. Healing at the same temperature for 50 h (Fig. S1a) generated sufficient oxide to adequately fill and bond the crack (Fig. 2d). Several unrepaired defects remained, but had a maximum size of approximately 2 μm, and were thus believed to be sufficiently small considering the size of pre-existing embedded flaws. Notably, the unrepaired defects shrank as the oxide increased in amount and dispersed into gaps (Fig. 2c,d), supporting the hypothesis that the healing material viscosity was low enough to fill irregularly shaped gaps, without stressing crack edges.

Although alumina is expected to promote SiO_2_ crystallisation, trace amounts of crystals were difficult to detect by X-ray diffraction (Fig. S1b). However, high-resolution transmission electron microscopy confirmed that the healing material was uniformly crystallised in healed cracks (Fig. 2e–g). In addition, fast Fourier transform patterns of a healed site were consistent with a single SiO_2_ cristobalite crystal (Fig. 2h) sized at least approximately 2 μm between the SiC layer and the adjacent Al_2_O_3_ matrix. This crystal grew in a similar orientation as SiC crystals (Fig. 2f), suggesting that the cristobalite nucleated at the SiC surface and grew epitaxially from the interface between SiC and the supercooled melt.

A second crystal was also detected at the interface with Al_2_O_3_ (Fig. 2g). Fast Fourier transform patterns were consistent with mullite (Fig. 2i), an intermediate compound of SiO_2_ and Al_2_O_3_ (Fig. 2j). The crystal formation highlights the excellent compatibility between SiC and Al_2_O_3_, and is likely a key element in the recovery of resistance to high temperatures. This crystal likely precipitated on the interface with alumina because the concentration of Al_2_O_3_ in the aluminosilicate supercooled melt increased as the cristobalite grew to the alumina surface, consistent with the fact that the oxide produced by reaction (1) contains a trace quantity of Al_2_O_3_ before crystallisation.

### Design for the 3D network of healing activator

**Figure 3:**
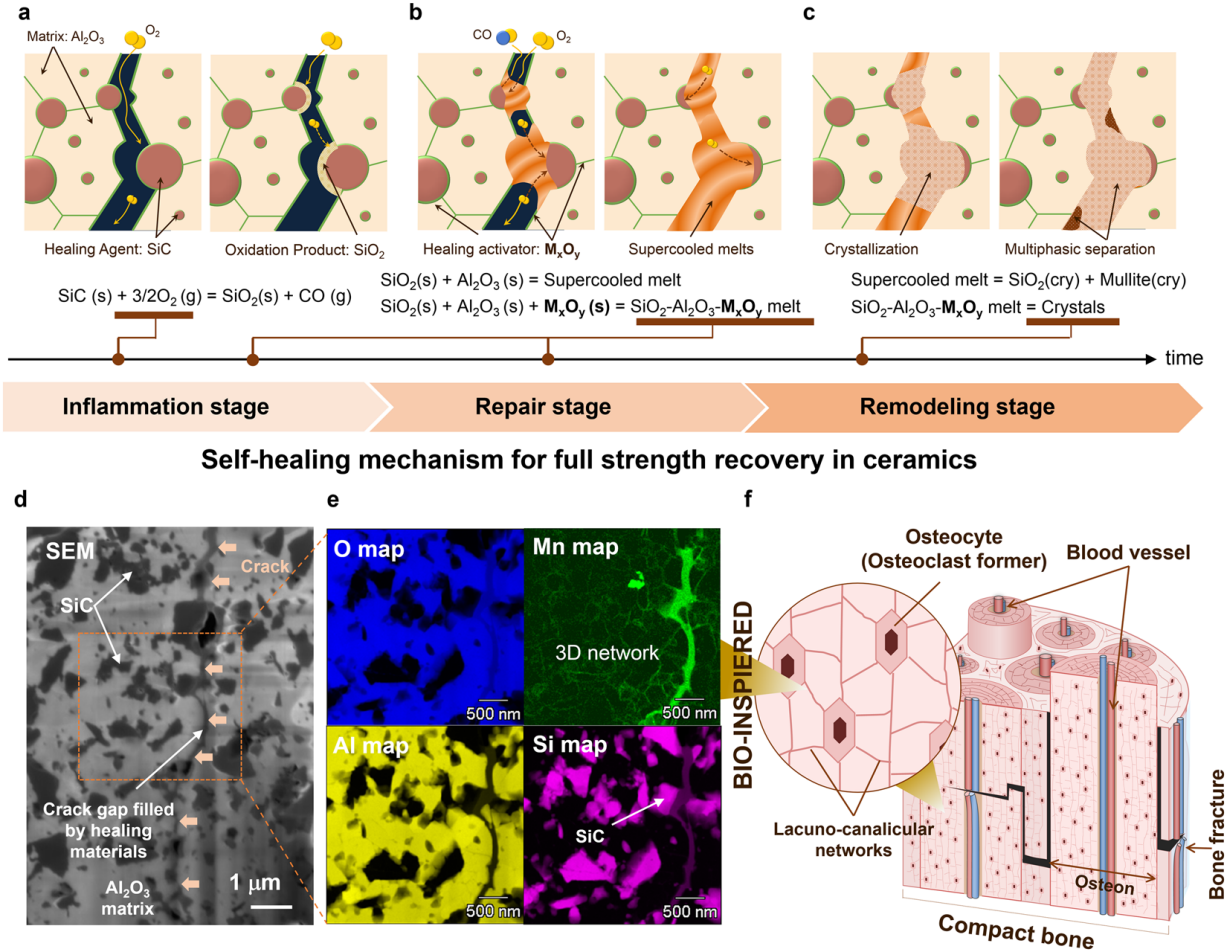
Self-healing in Al_2_O_3_/SiC composites and effect of healing activator network. (**a**) Oxygen penetrates cracked surfaces, and oxidizes SiC to SiO_2_ (defined as the inflammation stage). (**b**) Al_2_O_3_ and M_x_O_y_ dissolve into SiO_2_ to form a mechanically weak, low-viscosity supercooled melt, which completely fills irregularly shaped gaps (defined as the repair stage). (**c**) Mechanically strong crystals nucleate and grow in the supercooled melt (defined as the remodelling stage). (**d**) Scanning electron micrograph of an indentation crack filled by healing agent in a ceramic doped with 0.2 vol.% MnO and healed at 1,273 K for 1 h. (**e**) Mn-rich healing activator network bio-inspired by lacuna-canalicular networks. (**f**) Compact bone with networks of blood vessels and lacuna-canaliculi containing osteocytes.

The self-healing mechanism clearly indicates that dissolution of Al_2_O_3_ into SiO_2_ is critical both to efficiently fill gaps with low-viscosity supercooled melt and to deposit reinforcing crystals that contribute to full strength recovery. Inspired by bone healing, we divided this mechanism into three main stages, defined as inflammation, repair, and remodelling stages, respectively (see Fig. 3a–c). Thus, by appropriately designing and incorporating a healing activator that promotes these processes, it is possible to further enhance self-healing.

Key to the efficient formation of a mobile healing phase is to localise a small amount of healing activator to the fracture surface. As noted, cracks in Al_2_O_3_/30 vol.% SiC propagate mainly along the Al_2_O_3_ grain boundary and Al_2_O_3_/SiC interface (also see Fig. 3d,e). Thus, we attempted to create, using conventional liquid sintering, an initial microstructure that contains a 3D network of healing activator at the boundary and interface. Element mapping clearly showed that concentrated Mn at the fracture surface efficiently reacted with SiO_2_-Al_2_O_3_ melts and formed homogeneous Mn-rich healing materials in fracture gaps healed at 1,273 K for 1 hour (Fig. 3d,e). The formed healing material contained, on average, 76.71 mol% SiO_2_, 21.00 mol% Al_2_O_3_, and 2.28 mol% MnO. In addition, we designed the activator network to have a shorter period than the inter-particle spacing of SiC (Figs 3d,e and S2a,b) to enhance the reaction with SiO_2_ and to promote the formation of a homogeneous Mn-rich healing material. Our idea of a 3D network containing the healing activator was inspired by the lacuno-canalicular network containing osteocytes in bone structure (Fig. 3f).

In the initial stage of bone healing, blood fills gaps and elicits an inflammatory reaction to generate a hematoma, which is required to deposit temporary bone in the subsequent repair stage. Bio-inspired by blood and microvascular networks, Toohey^15^ proposed a self-healing polymer with liquid-phase healing agent networks. However, the liquid phases should not be incorporated into the initial structure of materials used at high temperatures to prevent significant loss of strength. Therefore, self-healing ceramics were originally designed to use atmospheric oxygen as a ‘mobile phase’ during high-temperature ‘inflammation’ (Fig. 3a), with the healing agent SiC distributed similarly to blood vessels. However, gap filling by only the gas phase was inefficient. In contrast, a blood-like supercooled melt with an activator (or Al_2_O_3_) can spread from healing agent to matrix, leading to efficient filling of fracture gap at high temperatures.

In addition to the efficient filling by low viscos melts, the blood-like supercooled melt rapidly delivers elements necessary for the repair stage (Fig. 3b). Accordingly, gap filling by the melts is eventually determined by the rate of SiC oxidation, which is limited by the diffusion of oxygen in the supercooled melt and by the escape of the CO produced. The reduction in viscosity due to the incorporation of healing activator (or Al_2_O_3_) is likely to substantially improve oxygen diffusion and CO escape. Although quantitative data on oxygen diffusion into supercooled melts are limited, a Stokes–Einstein relationship has been qualitatively demonstrated^33,34^. Also it has been reported that weight gain by isothermal oxidation of SiC increases in the presence of impurities which possibly decrease the viscosity of formed oxide layer^30,31^. In this manner, we developed self-healing high-temperature ceramics enabling efficient delivery of oxygen.

Finally, an activator (or Al_2_O_3_) with a 3D network structure breaks down the structure of strong glass SiO_2_ and promotes the subsequent crystallization and rebuilding of a robust healing material in the fracture gap (Fig. 3c). Collectively, these processes resemble bone repair and remodelling, during which osteoclasts resorb weak temporary bone and osteoblasts then remodel the tissue to generate a stronger structure^11,12^. Molecular rearrangements due to external forces do not occur in self-healing ceramics; however, numerous similarities remain.

### Selection of a healing activator

**Figure 4:**
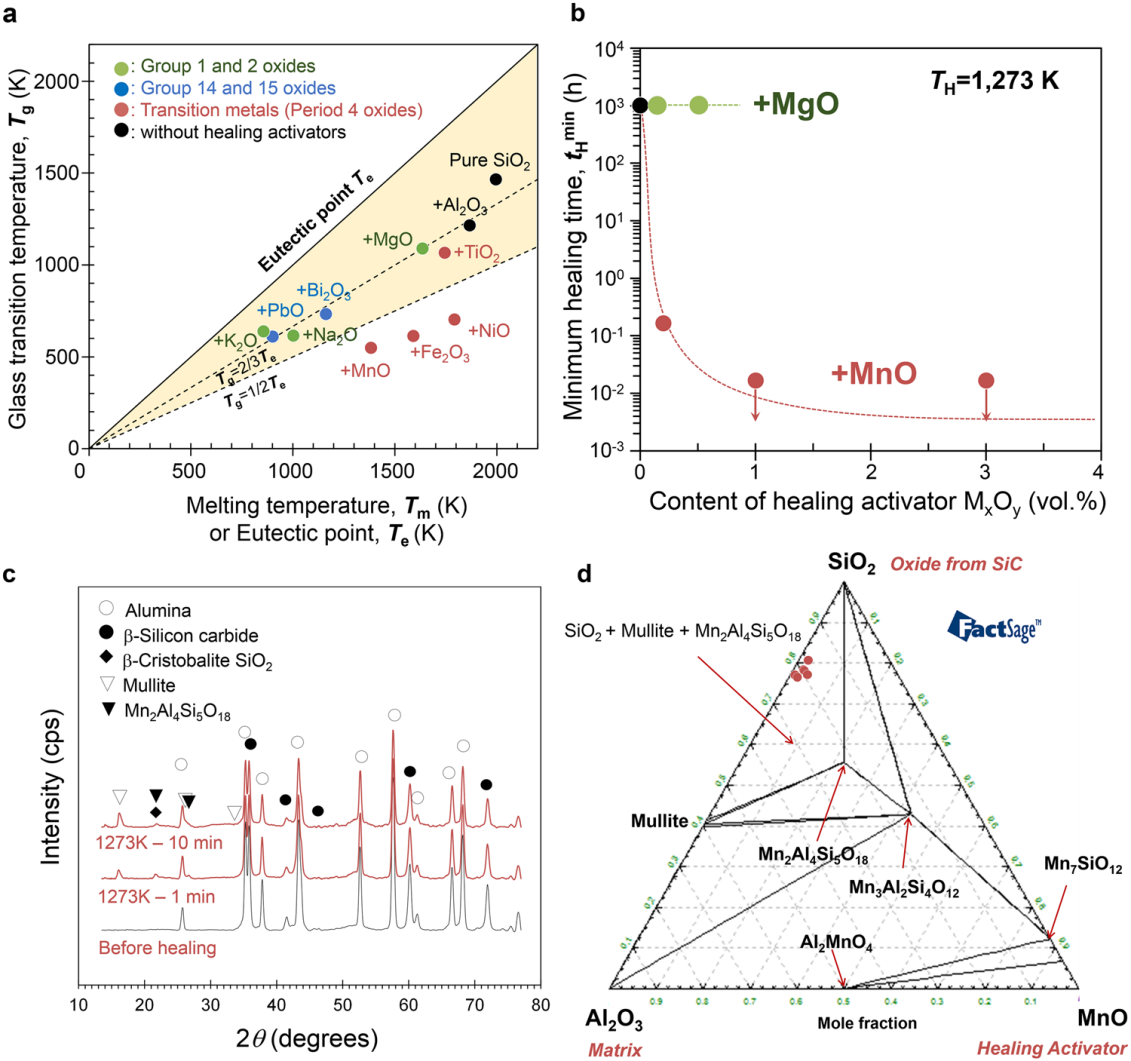
Selection of healing activator. (**a**) Glass transition temperature *T*_g_ calculated using FactSage thermodynamic software based on the composition of SiO_2_-Al_2_O_3_-M_x_O_y_ glass at the lowest eutectic point *T*_e_ (see Supplementary Materials). The reported *T*_g_ and *T*_e_ for Bi_2_O_3_ are also plotted for comparison^48^. (**b**) Effect of the healing activator M_x_O_y_ on the minimum time required to complete healing in Al_2_O_3_/SiC composites. (**c**) Representative X-ray diffraction patterns on the surface (not in gaps) before and after healing of Al_2_O_3_/SiC ceramics doped with 1.0 vol.% MnO. (**d**) Ternary phase diagram of Al_2_O_3_ (matrix) – SiO_2_ (oxide from SiC) – MnO (healing activator) system at 1,273 K estimated using FactSage. Red circle indicates the composition of the formed healing material in Fig. 3b measured by a scanning transmission electron microscope with dual energy dispersive X-ray spectrometry detectors.

An optimal healing activator was selected based on the glass transition temperature *T*_g_ of the resulting healing material, an indicator of SiO_2_-Al_2_O_3_-M_x_O_y_ glass viscosity, as well as the eutectic point *T*_e_ after crystallization, an indicator of temperature capability (Fig. 4a). *T*_*g*_ and *T*_*e*_ correspond to the lowest temperature at which healing with a supercooled healing melt occurs and the highest temperature at which such a melt hardens and solidifies, respectively. *T*_g_ and *T*_e_ were estimated for aluminosilicates (SiO_2_-Al_2_O_3_) doped with metal oxides M_x_O_y_ from families 1, 2, 14, and 15 and period 4 of the periodic table. In particular, SiO_2_-Al_2_O_3_-M_x_O_y_ compositions with the lowest eutectic points were selected for this estimation (Fig. S3), although whether equilibrium is achieved during rapid self-healing remains an open question.

M_x_O_y_ healing activators significantly decreased the *T*_g_ of aluminosilicate (~1,240 K). The *T*_*g*_/*T*_*e*_ ratio is empirically measured for various oxide phases and is between 1/2 and 2/3^35–37^. A similar trend was noted in values estimated by FactSage for various M_x_O_y_-doped healing materials. As shown in Fig. 4a, healing materials doped with the period 4 oxides MnO, Fe_2_O_3_, and NiO had an estimated *T*_*g*_/*T*_*e*_ of ~1/2, and were thus anticipated to be excellent healing activators. In particular, the *T*_*g*_ of MnO-doped materials was approximately 550 K, which is 667 K lower than that of aluminosilicate. Accordingly, MnO would decrease the viscosity of the healing material at 1,273 K from 1.57 × 10^10^ Pa·s to 4.40 × 10^1^ Pa·s (Fig. S3b) and thereby enhance the diffusion of oxygen into the supercooled melt to ultimately accelerate healing (Fig. 1a). Oxides of alkali metals (family 1) and alkali earth metals (family 2), including Na_2_O, K_2_O, and MgO, are frequently used to sinter alumina and also significantly decreased the estimated *T*_g_. However, the estimated *T*_g_/*T*_e_ was 2/3, implying that these materials are less favourable than those doped with MnO. In contrast, family 14 and 15 oxides, such as PbO and Bi_2_O_3_, markedly decreased the estimated *T*_*e*_ to 610 K and 733 K, respectively, and thus cannot be used at higher temperatures.

Even small quantities of an appropriate healing activator significantly accelerated self-healing (Fig. 4b). The required time to complete healing, *t*_*min*_, decreased as the amount of MnO increased, ultimately stabilising at 1.0 vol.% MnO and higher. In this manner, *t*_*min*_ was reduced by as much as 60,000 fold. In contrast, *t*_*min*_ was comparable in healing materials doped with MgO up to 0.5 vol.% as the viscosity of SiO_2_-Al_2_O_3_-MgO glass at 1,273 K is similar to that of aluminosilicate (Fig. S3b).

The addition of a healing activator also promoted the crystallisation of the healing materials (Fig. 4c). For example, doping with 1.0 vol.% MnO generated oxide crystals detectable by X-ray diffraction even after an abbreviated healing period. Indeed, SiO_2_ (cristobalite), mullite, and Mn_2_Al_4_Si_5_O_13_ crystals were observed on the surface after healing at 1,273 K for 1 and 10 min. These crystal phases coincided with the stable phase for measured compositions in the SiO_2_-Al_2_O_3_-MnO ternary phase diagram at 1,273 K estimated using FactSage (Fig. 4d). Thus, we concluded that the same crystals are also present in narrow gaps. As a general tendency, it is known that the addition of elements that decrease *T*_g_ can promote crystallisation of melts^38^, although it is unclear in this case why MnO promotes crystallisation. Further studies based on atomistic dynamic simulations^39^ will be helpful for understanding the role of MnO addition.

A healing activator must be carefully selected based on the intended application and operating environment. Our approach should facilitate activator selection and is suitable for many types of self-healing ceramics that combine an oxide matrix with a non-oxide healing agent.

### Innovation by a new design approach

**Figure 5:**
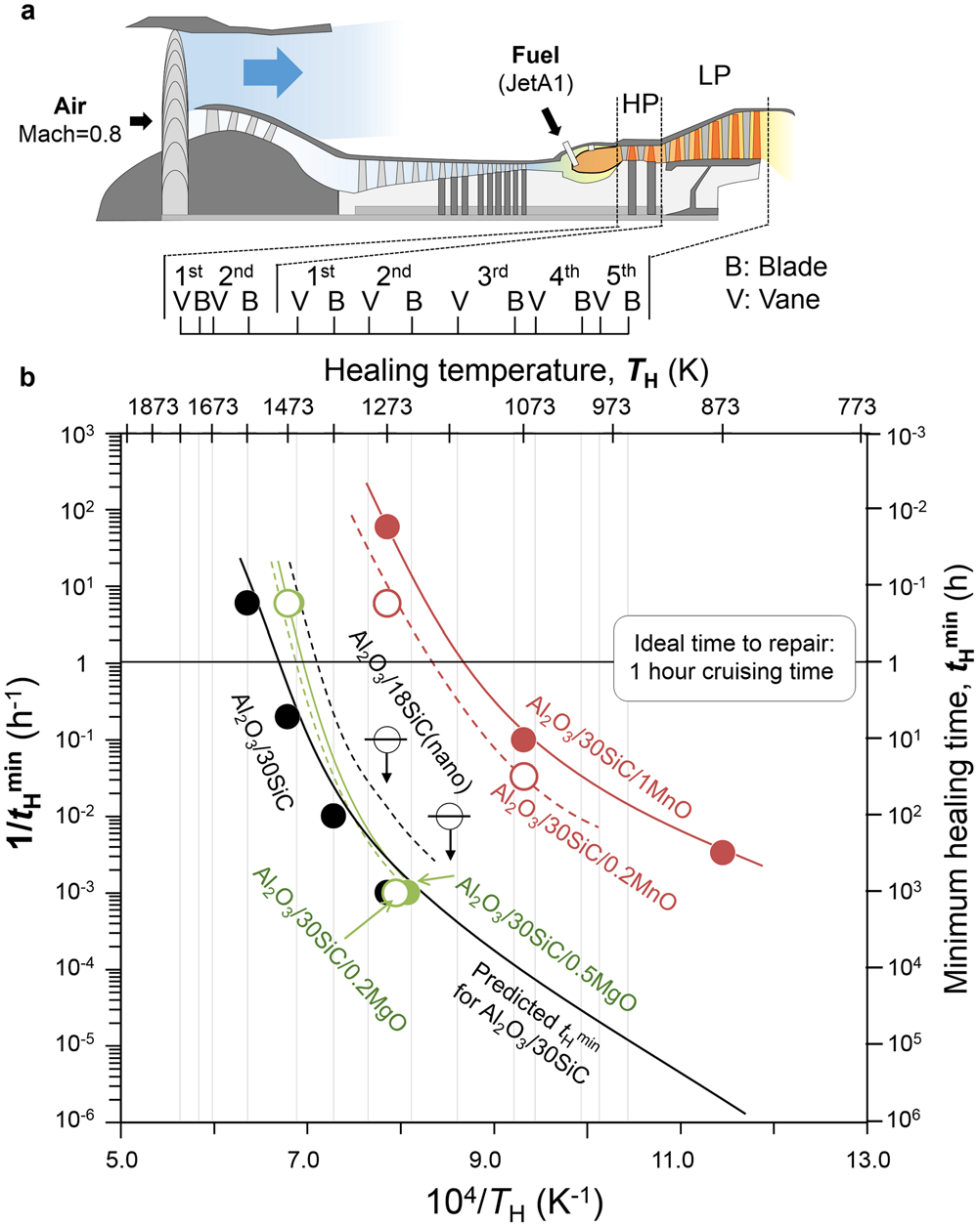
Simulated performance of self-healing ceramics in turbine blades. (**a**) Cross-section of a conventional CF6 jet engine, and the estimated gas temperature at each turbine blade and vane operating at TIT = 1,773 K without cooling^40^, and (**b**) temperature dependence of the minimum time to complete healing, *t*_min_, in ceramics with various types and concentrations of healing activator. For comparison, reported experimental data for alumina containing nanosized SiC particles^25,42^ are plotted. Center-lined symbols indicate that the specimen fractured the healed parts.

Our design strategy may be used to develop new self-healing ceramics suitable for high- or low-pressure stationary and rotary turbine blades^40^ operating over a range of temperatures (Fig. 5a,b). In particular, we found that MnO greatly reduces the required healing temperature, and that composite ceramics doped with 1.0 vol.% MnO completely self-heal at 1,273 K, 1,073 K, and 873 K in 1 min, 10 h, and 300 h, respectively. We note that *t*_*min*_ for MnO-free ceramics at 1,073 K and 873 K are extremely large and difficult to measure, but can be estimated^41^ from high-temperature oxidation of hot-pressed SiC containing a trace quantity of Al_2_O_3_. Thus, MnO-free ceramics were estimated to heal at these temperatures in 10,000 h (416 days) and 670,000 h (76 years), respectively, and are thus impractical to use. Similarly, MgO-doped materials self-heal more quickly than MgO-free materials, even at 1,473 K, which is already higher than the *T*_g_. Assuming that a ceramic turbine blade will remain reliable if a microscopic surface crack can be completely healed within 1 h while cruising, materials doped with 1 vol.% MnO, 0.2 vol.% MnO, or 0.5 vol% MgO would likely heal well in a 3^rd^-stage stationary blade, a 2^nd^-stage rotary blade, and a 1^st^-stage stationary blade in a low-pressure turbine.

Microstructure optimisation, especially by nanosizing of SiC^25,42^, is also useful for activating the inflammation stage (see Fig. 3a) at a lower healing temperature. For comparison, the reported data for alumina including nano-sized SiC particles with an average size of 10–30 nm are plotted in Fig. 5b. Our approach of accelerating repair and remodelling stages shows a much larger effect than a nanosizing approach. Although alumina including nano-sized SiC particles can exhibit rapid strength recovery, cracks in these materials cannot be healed completely at 1,173 K and 1,273 K, which are lower than and close to the *T*_g_ of aluminosilicate (~1,240 K), respectively, resulting in fracture from the healed parts^42^. Furthermore, excessive nanosizing reduces the strength recovery rate^25^. Thus, the efficient formation of a mobile supercooled melt during the repair stage (Fig. 3b) is of key importance for full strength recovery by self-healing. Additionally, it is important to note that two design approaches for accelerating deferent healing stages can coexist. Thus, doping of a healing activator in materials will be further accelerate the healing rate and reduce the required reaction temperature by microstructural optimization with nanosizing of the healing agent.

Our new design approach will be key for application of the ceramic matrix composite to rotary blades with stringent safety requirements, which could possibly be damaged by the unpredictable impact of foreign objects, and will meet the requirements in aircraft engines^43^. However, flight conditions and the rate of crack propagation are also critical factors. Naturally, the use of these ceramics would require further improvements to ensure sufficient time for healing, perhaps by incorporating biomimetic reinforcing structures, such as fibres^1,3,7,8^ and shells^4–6^, and by slowing the rate of crack propagation.

### Concluding remarks

A bio-inspired design that incorporates a 3D network of healing activator markedly enhances self-healing and reduces the required healing temperature, resulting in materials that retain structural integrity, despite damage that would be catastrophic in brittle materials. The flexible selection method based on thermodynamics also identifies the optimal healing activator for the required operating temperature, regardless of matrix and healing agent. We are currently evaluating the high-temperature mechanical properties of the materials described here and investigating ways to incorporate a reinforcing hierarchical structure similar to that in human bone.

## Methods

### Preparation of composite materials with a 3D network of healing activator

α-Al_2_O_3_ (99.99% pure, AKP-50; Sumitomo Chemicals, Japan) and α-SiC (Ultrafine; Ibiden, Ogaki, Japan) had average diameters of 0.5 μm and 0.27 μm, respectively, whereas the healing activators MnO (99.9% pure; Kojundo Chemical Laboratory, Saitama, Japan) and MgO (99.9% pure; Xuancheng Jingrui New Material, Xuancheng, China) had average diameters of 5–10 μm and 0.25 μm, respectively.

Using an alumina ball and a mill pot, a suspension of Al_2_O_3_ (average diameter 0.5 μm) and 30 vol.% SiC (average diameter 0.27 μm) was thoroughly ball-milled in alcohol for 24 h with or without 0.2–3.0 vol.% MnO or MgO. To form an M_x_O_y_-rich phase at the grain boundary, Al_2_O_3_/SiC composites with MnO or MgO were sintered for 1 h under Ar at 40 MPa and at 1,823 K and 1,973 K, respectively. As the eutectic point for MnO-Al_2_O_3_ is 1,728 K, MnO-doped composites were sintered in the liquid phase, as is typically done. However, MgO-doped composites^44^ were sintered below the eutectic point for MgO-Al_2_O_3_, i.e. 2,253 K.

### Indentation, self-healing, and bending test

Sintered plates were cut into 3 mm × 4 mm × 22 mm bars and polished to mirror finish. A semi-elliptical surface crack of 110-μm length was introduced at the centre of the specimen by Vickers indentation, using a load of 19.6 N. The aspect ratio of the crack (*a*/*c*) was 0.9, where *a* and *c* represent the depth and half of the crack length, respectively. The fracture toughness was obtained by the indentation-fracture (IF) method according to JIS standard^45^. Cracked specimens were heated at 873–1,573 K for 1 min to 1,000 h in air. Finally, healed specimens were tested by three-point bending at room temperature over a span length of 16 mm (see Fig. 1).

### Microstructure of healed cracks

The 3D structure of a healed crack was analysed on an orthogonally arranged, focused ion beam-scanning electron microscopy system^46^ (SMF-1000; Hitachi High-Tech Science, Tokyo, Japan). Briefly, healed surfaces near the indentation tip were ion-milled with a slice step size of 10 nm using a Ga^+^ beam at an acceleration voltage of 30 kV. At each slice, 1,000 cross-sectional images (10 × 10 μm) were collected using an electron beam at an acceleration voltage of 10 kV. Subsequently, these images were used to reconstruct a 4 × 3 × 2.5 μm 3D volume using ImgToVol (Cybernet Systems, Ann Arbor, MI, USA), MicroAVS 19.0 (Cybernet Systems), and ImageJ (NIH, Bethesda, MD, USA).

After imaging, a thin 10 × 10 × 0.2 μm plate was fixed on a semi-circular mesh, and thinned to below 50 nm using a low-energy focused Ar ion beam (Model 1040 NanoMill; Fischione Instruments, Export, PA, USA)^47^. The thinned sample was then imaged on a high-resolution transmission electron microscope (JEM-3100FEF; JEOL, Tokyo, Japan) at an accelerating voltage of 300 kV. Crystal structures in the narrow fracture gaps were identified by fast Fourier transform imaging in DigitalMicrograph (Gatan) Furthermore, the sample was also imaged on a scanning transmission electron microscope with dual energy dispersive X-ray spectrometry detectors (JEM-2800; JEOL) for identifying the localisation of the activator.

The phase compositions and crystal structure of the healed bending bar specimens were also identified with X-ray diffraction (D8 Discover) using CuKα radiation generated at 40 kV and 40 mA.

### Selection of healing activator

To select the optimal activator to accelerate self-healing, the eutectic point for materials doped with M_x_O_y_ was calculated in FactSage 7.0 according to thermodynamic principles, and compared with experimental bubbling temperatures observed by high-temperature *in situ* microscopy. Additionally, viscosity and glass transition temperatures were estimated using FactSage.

## Electronic supplementary material

Supplementary Materials
