# Supplementary material for: A Novel Design Approach for Self-Crack-Healing Structural Ceramics with 3D Networks of Healing Activator
Source: Sci Rep. 2017 Dec 19;7:17853. doi: 10.1038/s41598-017-17942-6 (PMC5736619; doi:10.1038/s41598-017-17942-6)
Supplement: Supplementary file 1 — Supplementary Materials [file 41598_2017_17942_MOESM1_ESM.pdf]

Supplementary Materials for

A Novel Design Approach for Self-Crack-Healing Structural Ceramics with 3D Networks of Healing Activator

Toshio Osada[1,*], Kiichi Kamoda[1,3], Masanori Mitome[2], Toru Hara[1], Taichi Abe[1], Yuki Tamagawa[1,3], Wataru Nakao[3] & Takahito Ohmura[1]

[1]National Institute for Materials Science, 1-2-1 Sengen, Tsukuba, Ibaraki 305-0047, Japan. [2]National Institute for Materials Science, 1-1 Namiki, Tsukuba, Ibaraki 305-0044, Japan. [3]Yokohama National University, 79-5 Tokiwadai, Hodogaya, Yokohama 240-8501, Japan. [*]Correspondence and requests for materials should be addressed to T.O. (email: OSADA.Toshio@nims.go.jp.

This PDF file includes:

Method

Supplementary text and references

Figures S1, S2, S3

Methods

Selection of healing activator. To select the optimal activator to accelerate self-healing, the eutectic point for materials doped with M_xO_y was calculated in FactSage 7.0 according to thermodynamic principles, and compared with experimental bubbling temperatures observed by high-temperature *in situ* microscopy. Additionally, viscosity and glass transition temperatures were estimated using FactSage.

High-temperature *in situ* microscopy and bubbling. To confirm the decrease in viscosity and the incorporation of the healing activator into the healing agent, we directly visualized bubbling[S1] using high-temperature *in situ* microscopy (Yonekura, Osaka, Japan). Materials were heated to 1,273 K at 50 K/min, and at 20 K/min thereafter, and the temperature at which a CO gas bubble was rapidly formed through the supercooled melt was considered the bubbling temperature T_B.

Supplementary text

Strength recovery

Al_2O_3/SiC composites self-healed completely between 1,273 K and 1,573 K (**Fig. S1**). The rate of strength recovery, as well as the minimum time to complete healing, t_{min}, are

mostly determined by the rate of oxide production, which, in turn, is determined by temperature. For example, t_{min} is 100 h and 1,000 h at 1,373 K and 1,273 K, respectively. Thus, self-healing below 1,373 K is impractical. Furthermore, crystallized oxidation products were undetectable by X-ray diffraction after healing at 1,473 K for 1, 5, and 50 h. However, a weak peak consistent with β-cristobalite SiO_2 was detected on surfaces healed at 1,273 K for 1,000 h. Meanwhile, the detected cristobalite corresponds a reported stable crystal at temperature above approximately 1,273 K[S2]. As amount of cristobalite was slight, we found that high-resolution transmission electron microscopy was more useful to detect crystals.

Comparison of initial microstructures and crack gap filling behaviour.

Materials with or without MnO are similar in SiC content and particle size, particle size distribution, and alumina grain size (see **Fig.S2a, b**), and they have similar mechanical properties such as bending strength and fracture toughness. The two materials differ in the presence of Mn-rich networks in the initial microstructure and crack-gap filling rate. It is clear that the material with MnO can fully fill the crack-gap after healing even at 1,273K for 1 hour (**Fig. S2a**), which is 200 K lower than the healing temperature of the MnO-free material (**Fig. S2b**). High-resolution transmission electron microscopy

inside the healed material with MnO confirmed that crystalline structures with a nanometre width were present on the Mn-rich phase on the Al_2O_3 grain boundary (**Fig. S2c, d**). In addition, fast Fourier transform patterns of the selected area of (e), (f) and (g) in **Fig. S2d** were consistent with those of a MnO cubic crystal (**Fig. S2e**), corundum alumina with [12 9 3] tilt, and corundum alumina with [0 3 9] tilt, respectively.

Furthermore, the ternary phase diagram of the Al_2O_3-SiO_2-MnO system at 1,273 K, which is the same as the healing temperature for the MnO-doped material, is shown in **Fig. 4d**. The diagram was estimated using FactSage. According to the phase diagram of Al_2O_3-MnO, MnO and Al_2MnO_4 crystals were estimated to be the thermodynamically stable phase. Thus, we concluded that the healing Mn-rich network was mainly composed of a MnO cubic crystal phase, although it was not possible to observe trace amounts of Al_2MnO_4 in this study. Additionally, the trace amounts of grain boundary phases including MnO and Al_2MnO_4 that were stable in the phase diagram (**Fig. 4d**) could not be detected by X-ray diffraction in the initial material with MnO (**Fig. 4dc**).

Healing material composition, viscosity, glass transition temperature, and eutectic point

Soon after a crack triggers passive oxidation of SiC (inflammatory stage) at high

temperatures, the healing activator M_xO_y and a small amount of Al_2O_3 dissolve, yielding SiO_2 and resulting in a low-viscosity supercooled SiO_2-Al_2O_3-M_xO_y glass. We note that doping with a small amount of healing activator accelerated the formation of the supercooled melt. Indeed, bubbling, as observed by *in situ* microscopy, occurred at a lower temperature in M_xO_y-doped ceramics (**Fig. S2a**), suggesting that M_xO_y and Al_2O_3 are rapidly dissolved into SiO_2. In addition, the observed bubbling temperature was consistent with the lowest eutectic points estimated from ternary phase diagrams deposited in FactSage 7.0. Based on these results, we estimated that supercooled melts contain a maximum of 51.9 mol% SiO_2, 8.2 mol% Al_2O_3, and 39.9 mol% MnO at the lowest eutectic point, although it is difficult to establish whether equilibrium is completely achieved at healing temperatures lower than the eutectic point. We note that the estimated concentration of M_xO_y in the supercooled melt is significantly higher than the initial concentration incorporated into the suspension (0.2 vol.%).

The dissolution of M_xO_y and Al_2O_3 into SiO_2 also significantly decreased the viscosity of the melt (**Fig. S2b**), which can be estimated at various temperatures in FactSage using the composition of the melt at the lowest eutectic point. Here, we only show the estimated and reported experimental viscosity for pure SiO_2 glass, SiO_2-Al_2O_3, and SiO_2-Al_2O_3 melts with MnO and MgO [S3,S4]. The decrease in viscosity also

decreased the glass transition temperature T_g. For common glasses, T_g is typically reported as the temperature at which the viscosity is $10^{11.3}$ Pa·s [S5] or 10^{13} Pa·s [S6]. In this study, we estimated T_g corresponding to $\eta = 10^{11.3}$ Pa·s. Thus, we used T_g as the lower temperature limit in selecting the healing activator, as discussed in the main text (**Fig. 4a**).

References

S1. Goto, T. & Homma, H. High-temperature active/passive oxidation and bubble formation of CVD SiC in O_2 and CO_2 atmospheres. *J. Euro. Ceram. Soc.* **22**, 2749–2756 (2002).

S2. Pagliari, L. *et al.*, A kinetic study of the quartz-cristobalite phase transition, *J. Euro. Ceram. Soc.* **33**, 3403–3410 (2013).

S3. Urbain, G., Bottinga, Y. & Richet, P. Viscosity of liquid silica, silicates and alumino-silicates. *Geochim. Cosmochim. Acta* **46**, 1061–1072 (1982).

S4. Toplis, M. J. & Dingwell, D. B. Shear viscosities of CaO-Al_2O_3-SiO_2 and MgO-Al_2O_3-SiO_2 liquids: Implications for the structural role of aluminium and the degree of polymerisation of synthetic and natural aluminosilicate melts. *Geochim. Cosmochim. Acta* **68**, 5169–5188 (2004).

S5. Shelby, J. E. Introduction to glass science and technology (*The Royal Society of*

Chemistry, UK, 2005).

S6. Sakka, S. & Mackenzie, J. D. Relation between apparent grass transition temperature and liquidus temperature for inorganic glasses, *J. Non-Cryst. Solids*. **6**, 145-162 (1971).

Supplementary figures

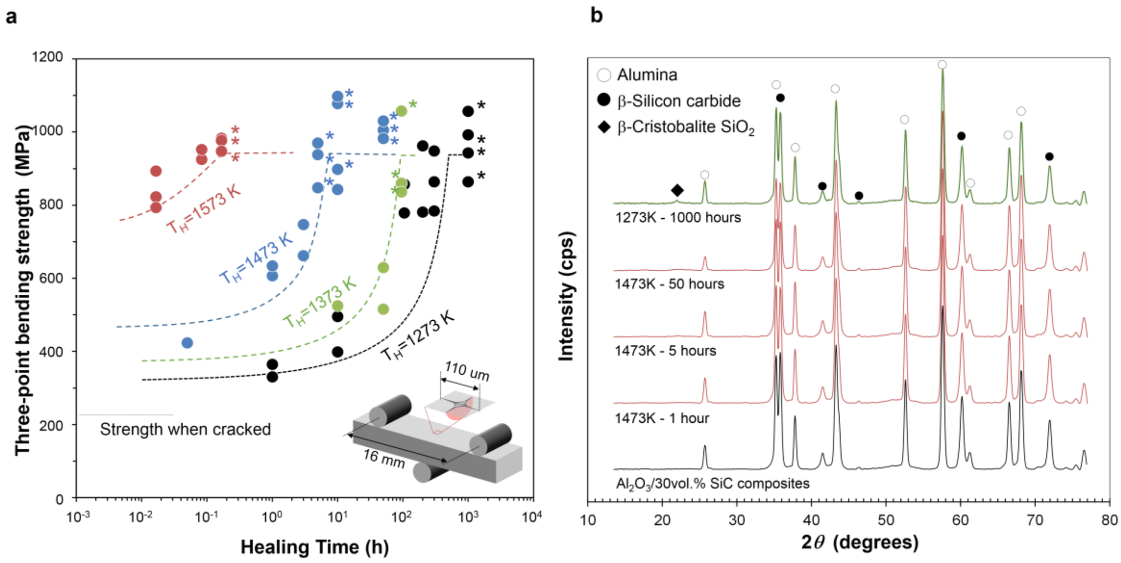

Figure S1. Self-healing ceramics without healing activator. (**a**) Self-healing and strength recovery of Al_2O_3/SiC composites healed at 1,273 K, 1,373 K, 1,473 K, and 1,573 K. (**b**) Representative X-ray diffraction patterns before and after healing. Crystallized oxidation products were undetectable after healing at 1,473 K for 1, 5, and 50 h; however, a small amount of ß-cristobalite SiO_2 was detected after healing at 1,273 K for 1,000 h.

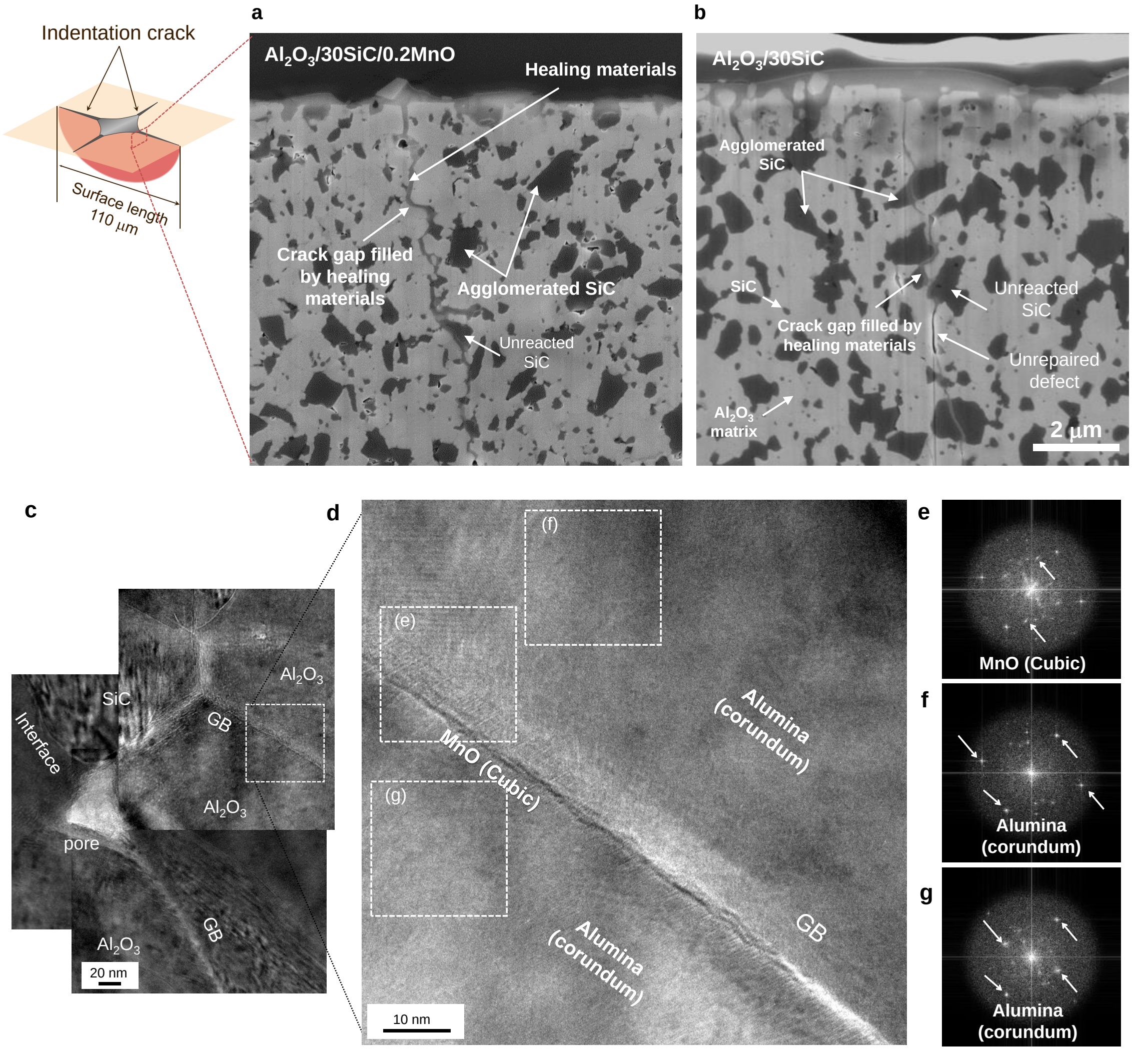

Fig. S2. Comparisons of microstructures and crack gap filling behaviour with and without healing activator at damage site close to indent tip. (**a**) Al_2O_3/SiC composites healed at 1,473 K for 50 hours. (**b**) Al_2O_3/SiC composites doped with 0.2 vol.% of the healing activator, MnO and healed at 1,273 K for 1 hour. (**c–g**) High-resolution transmission electron microscopy (**c**), grain boundary phase between Al_2O_3 and Al_2O_3 (**d**). Fast Fourier transform patterns indicating MnO (**e**), alumina (**f**), and alumina (**g**) at sites marked e, f, and g, respectively, in **d**.

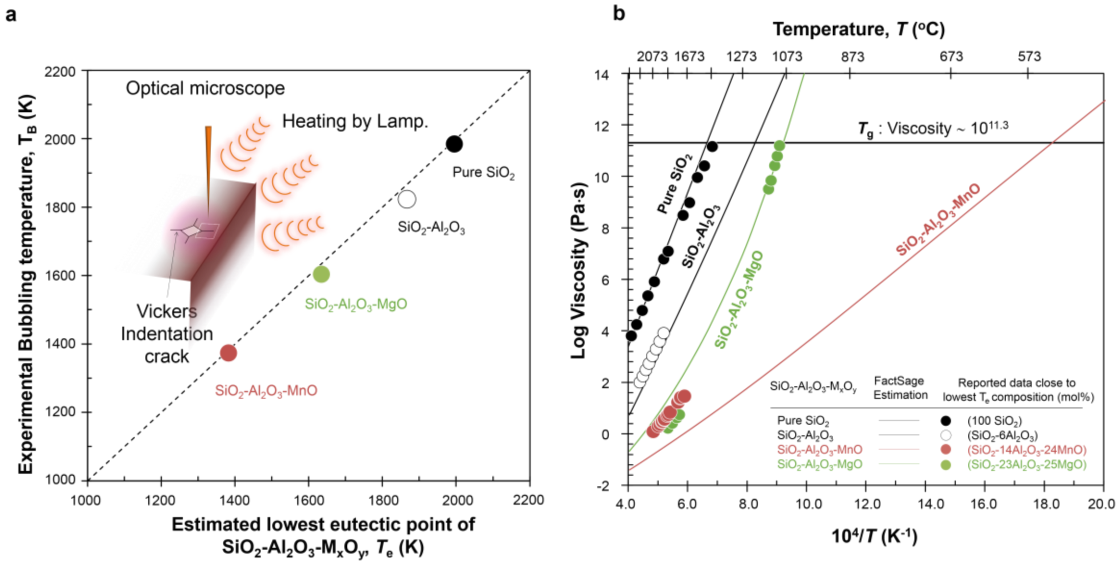

Figure S3. Self-healing ceramics with healing activator. (**a**) Relationship between bubbling temperature obtained by in situ microscopy and the lowest eutectic point estimated from FactSage for Al_2O_3/SiC doped with or without 0.2 vol.% MnO and 0.2 vol.% MgO. For comparison, pure SiO_2[S1] is also plotted. (**b**) Temperature dependence of the viscosity of the healing materials SiO_2-Al_2O_3-M_xO_y and pure SiO_2. Lines indicate viscosity estimated by FactSage for SiO_2 containing 4.9 mol% Al_2O_3 at the eutectic point, SiO_2 containing 8.2 mol% Al_2O_3 and 33.9 mol% MnO, and SiO_2 containing 10 mol% Al_2O_3 and 30 mol% MgO. For comparison, reported experimental data close to those at the lowest T_e are plotted for SiO_2 containing 6 mol.% Al_2O_3 (white circles) [S3], SiO_2 containing 14 mol.% Al_2O_3 and 24 mol.% MnO (red circles)[S4], SiO_2 containing 23 mol.% Al_2O_3 and 25 mol.% MgO (green circles) [S4], and pure SiO_2 (black circles) [S3]. We defined the glass transition temperature T_g as the temperature at which the viscosity equals $10^{11.3}$ Pa·s[S5].
